# Thoracoscopic and laparoscopic approach for pleuroperitoneal communication under peritoneal dialysis: a report of four cases

**DOI:** 10.1186/s40792-023-01635-6

**Published:** 2023-04-08

**Authors:** Teppei Hashimoto, Toshihiro Osaki, Soichi Oka, Takahisa Fujikawa

**Affiliations:** 1 Department of Thoracic Surgery, Kokura Memorial Hospital, 3-2-1, Asano, Kokurakita-ku, Kitakyuusyu-shi, Fukuoka, 802-8555 Japan; 2 Department of Gastroenterological Surgery, Kokura Memorial Hospital, 3-2-1, Asano, Kokurakita-ku, Kitakyuusyu-shi, Fukuoka, 802-8555 Japan

**Keywords:** Pleuroperitoneal communication, Thoracic and laparoscopic surgery, Continuous ambulatory peritoneal dialysis hydrothorax, Pneumoperitoneum, Laparoscopic approach

## Abstract

**Background:**

Pleuroperitoneal communication (PPC) is a rare complication of continuous ambulatory peritoneal dialysis (CAPD) and often forces patients to switch to hemodialysis. Some efficiencies of video-assisted thoracic surgery (VATS) for PPC have been reported recently; however, there is no standard approach for these complications. In this case series, we present a combined thoracoscopic and laparoscopic approach for PPC in four patients to better assess its feasibility and efficiency.

**Case presentation:**

Clinical characteristics, perioperative findings, surgical procedures, and clinical outcomes were retrospectively analyzed. We combined VATS with a laparoscopic approach to detect and repair the diaphragmatic lesions responsible for PPC. We first performed pneumoperitoneum in all patients following thoracoscopic exploration. In two cases, we found bubbles gushing out of a small pore in the central tendon of the diaphragm. The lesions were closed with 4-0 non-absorbable monofilament sutures, covered with a sheet of absorbable polyglycolic acid (PGA) felt, and sprayed with fibrin glue. In the other two cases without bubbles, a laparoscope was inserted, and we observed the diaphragm from the abdominal side. In one of the two cases, two pores were detected on the abdominal side. The lesions were closed using sutures and reinforced using the same procedure. In one case, we failed to detect a pore using VATS combined with the laparoscopic approach. Therefore, we covered the diaphragm with only a sheet of PGA felt and fibrin glue. There was no recurrence of PPC, and CAPD was resumed at an average of 11.3 days.

**Conclusions:**

The combined thoracoscopic and laparoscopic approach is an effective treatment for detecting and repairing the lesions responsible for PPC.

## Background

Pleuroperitoneal communication (PPC) is a rare condition, with an incidence of approximately 1.6–10%, often forcing patients undergoing continuous ambulatory peritoneal dialysis (CAPD) to switch to hemodialysis [1, 2]. Although the efficiency of video-assisted thoracic surgery (VATS) for PPC has been reported, to the best of our knowledge, no effective and definitive method is available to identify and repair diaphragmatic fistulas responsible for PPC.

In this case series, the feasibility and effectiveness of VATS combined with a laparoscopic approach for PPC were evaluated.

## Case presentation

From January 2019 to May 2022, we performed VATS combined with a laparoscopic approach for four patients with end-stage renal diseases with PPC during CAPD.

**Fig. 1:**
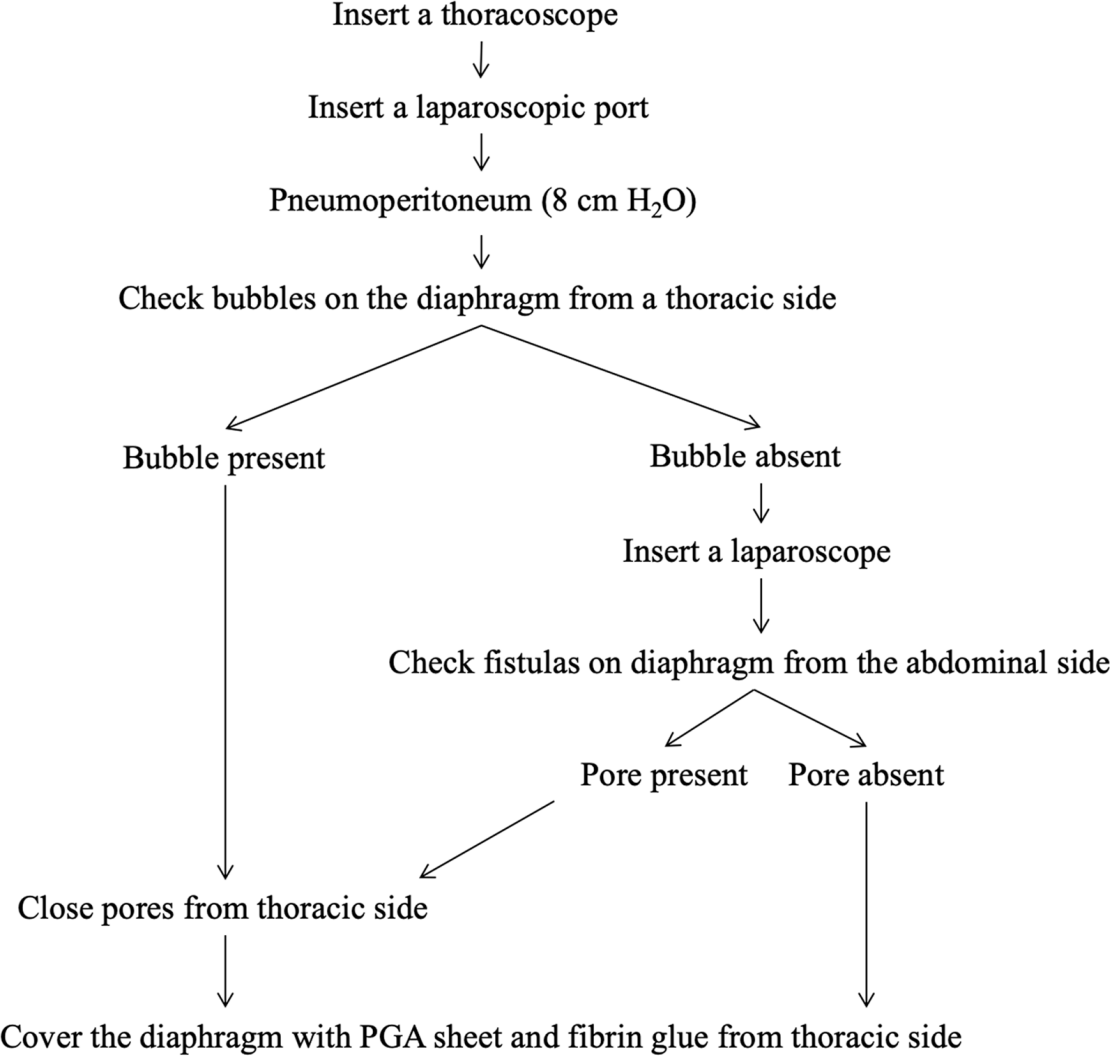
Surgical treatment strategy for pleuroperitoneal communication

Table 1 shows the patient characteristics. One of the patients was male, and three were female. The mean age was 69.8 years, and all PPCs occurred on the right side. The primary diseases leading to the introduction of CAPD were as follows: diabetic nephrosis in two patients, nephrosis in one patient, and renal sclerosis in one patient. The mean duration of CAPD before PPC was 154 days. The surgical treatment strategy is shown in Fig. 1.

**Table 1:** Patient characteristics, operative findings, and outcomes

Patient characteristics					Operative findings			Outcome	
Case	Age/Sex	Side	Renal disease	Duration of CAPD before PPC (days)	Bubble by pneumoperitoneum	Laparoscopic observation for fistulas	Procedure	CAPD resume (days)	PPC recurrence (Postoperative observation period)
1	58/F	Right	NS	11	Present	(No inspection)	Suture + PGA sheet + Fibrin	19	None (665 days)
2	76/F	Right	RS	124	Present	(No inspection)	Suture + PGA sheet + Fibrin	7	None (115 days dead by cancer)
3	67/M	Right	DN	168	None	None	PGA sheet + Fibrin	8	None (334 days)
4	78/F	Right	DN	313	None	Present	Suture + PGA sheet + Fibrin	8	None (198 days)

*CAPD* continuous ambulatory peritoneal dialysis, *PPC* pleuroperitoneal communication, *NS* nephrotic syndrome,

*RS* renal sclerosis, *DN* diabetic nephropathy, *PGA* polyglycolic acid

**Fig. 2:**
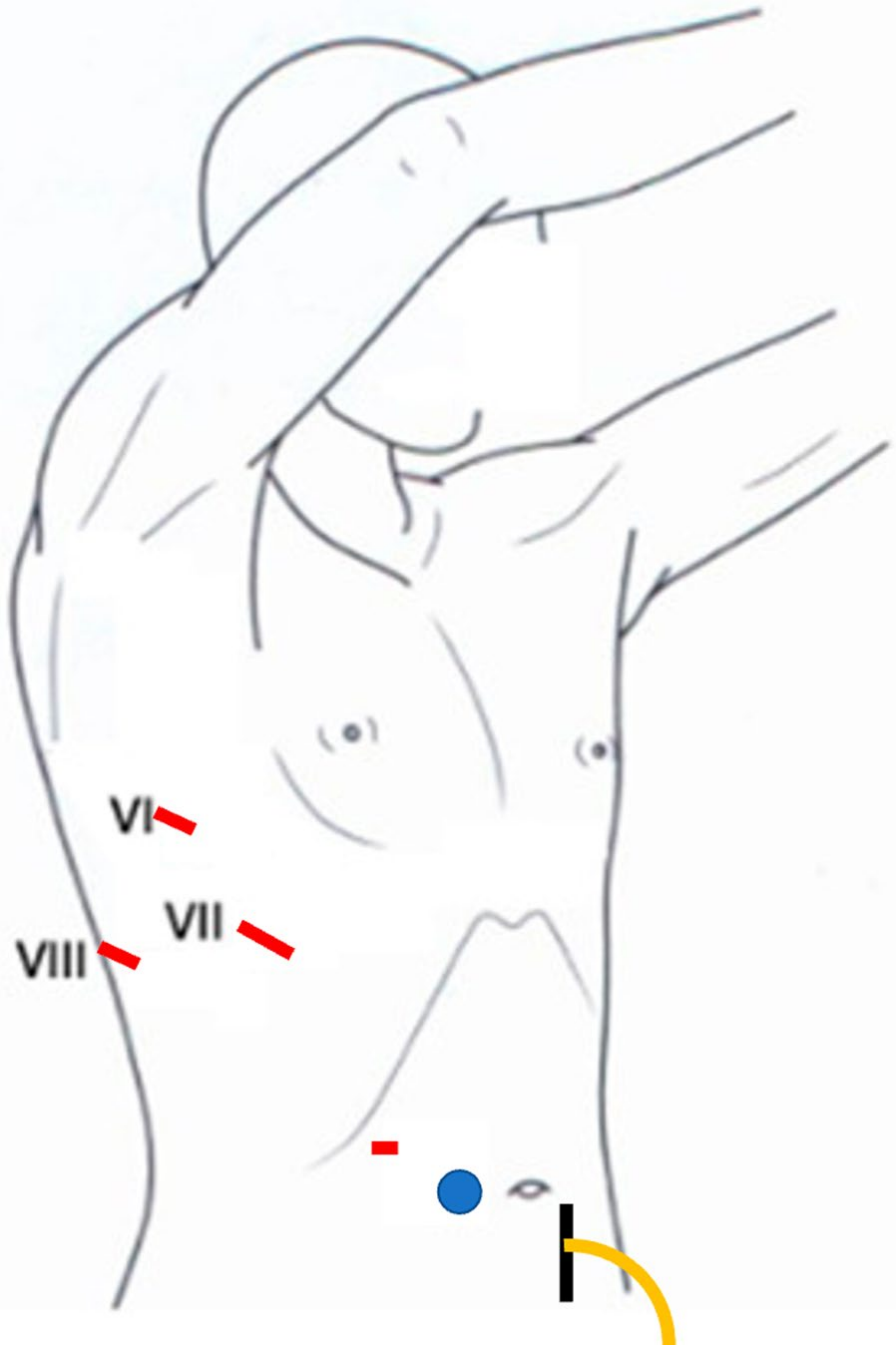
Intraoperative position of the patients and the thoracoscopic and laparoscopic ports. VATS in the left-sided half-lateral decubitus position; three 2-cm thoracic ports were created in the seventh, sixth, and eighth intercostal spaces on the anterior, middle, and posterior axillary lines, respectively. A thoracoscope was inserted in the middle axillary line and the other two ports were used to expand the operative fields. One skin incision was made to the right side of the navel, and a laparoscope was inserted into the port (blue round) because a 5-cm longitudinal incision had been made 17 cm cranially from the pubic bone and two lateral fingers to the left of the navel for the placement of the PD catheter (black vertical line). In cases without bubbles, we added an additional laparoscopic port under the right hypochondrium (red horizontal line) to carefully observe the diaphragm

Under general anesthesia, all patients were intubated with a double-lumen endotracheal tube and placed in a left-sided half-lateral decubitus position. Three 2-cm skin incisions were created in the seventh, sixth, and eighth intercostal spaces on the anterior, middle, and posterior axillary lines, respectively. A 30°, 10-mm thoracoscope was inserted in the sixth intercostal space of the middle axillary line. The other ports were used to expand the operative fields, and we carefully observed the diaphragm for fistulas. We then added a single laparoscopic port by open method, on the right side of the abdominal cavity, to avoid damaging the intestinal tract and prevent CAPD catheter infection (Fig. 2). At our hospital, a 5-cm longitudinal incision is created 17-cm cranially from the pubic bone and two-fingers lateral to the left of the navel for the placement of the PD catheter. When inserting the laparoscope, we checked for intestinal damage, and after confirming there was no intestinal damage, pneumoperitoneum was initiated at 8 cmH_2_O, and the bubbles were checked from the thoracic side. In cases without bubbles, we added another laparoscopic port under the right hypochondrium, and a laparoscope was inserted to inspect the diaphragm in the abdominal cavity. In cases in which fistulas were identified, the lesions were closed from the thoracic side with 4-0 non-absorbable monofilament sutures. Finally, in all cases, the lesions were reinforced with a sheet of absorbable polyglycolic acid (PGA) felt (Neoveil, Gunze, Osaka, Japan) and fibrin glue (Beriplast P, CSL Behring, King of Prussia, PA, USA).

**Fig. 3:**
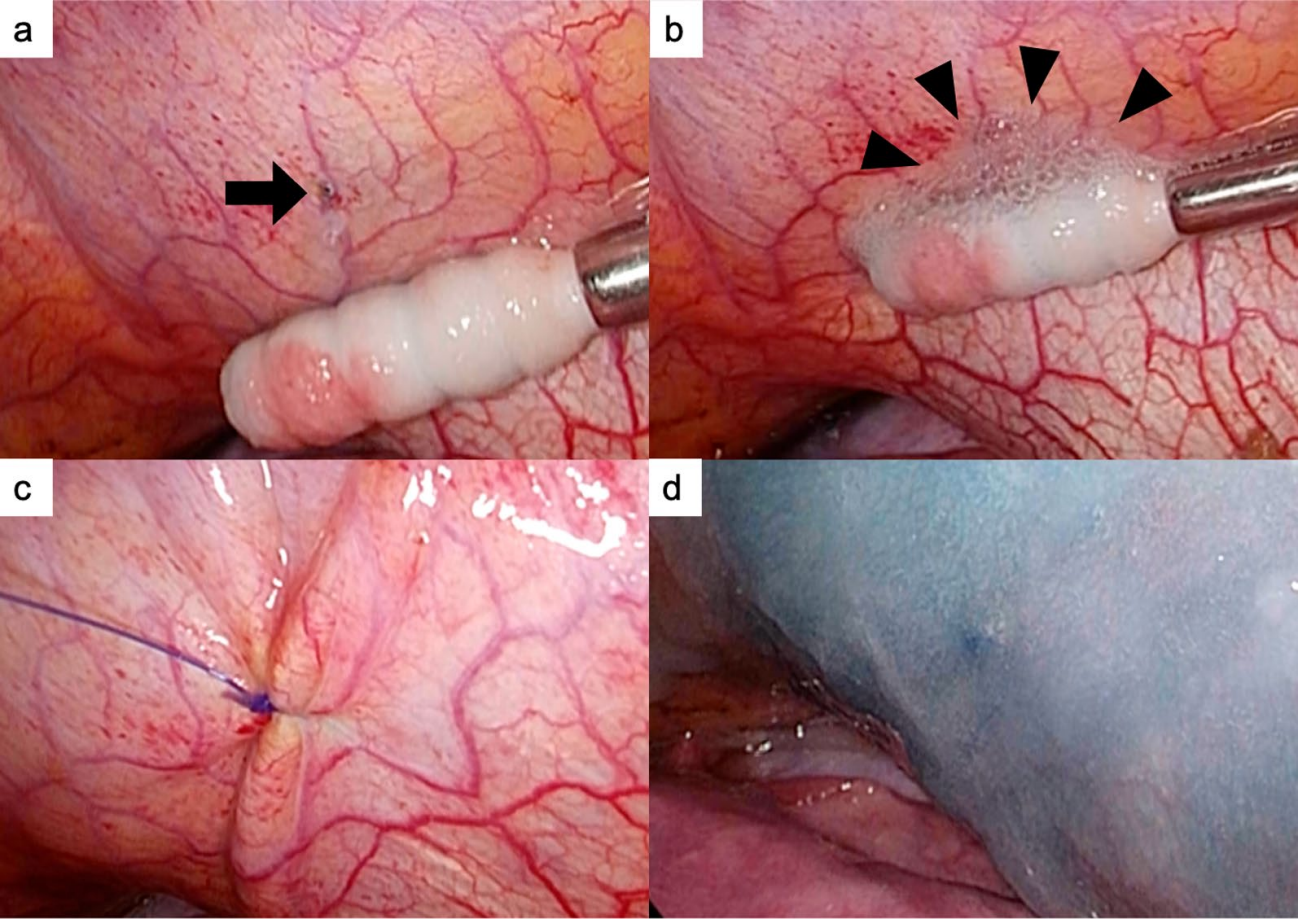
Surgical findings of case 1.** a** A pore was detected on the central tendon of the diaphragm with a thoracoscope (arrow). **b** Bubbles were observed in this small pore after pneumoperitoneum (arrowhead). **c** Small pores were closed with a single 4–0 non-absorbable monofilament Z suture. **d** The central tendon around the reinforcement was covered with a sheet of absorbable polyglycolic acid felt and sprayed with fibrin glue on the thoracic side

In cases 1 and 2, we could find bubbles gushing out of a small pore at the right central tendon of the diaphragm with a thoracoscope under pneumoperitoneum. The lesions were closed with sutures, covered with PGA sheets, and sprayed with fibrin glue (Fig. 3).

**Fig. 4:**
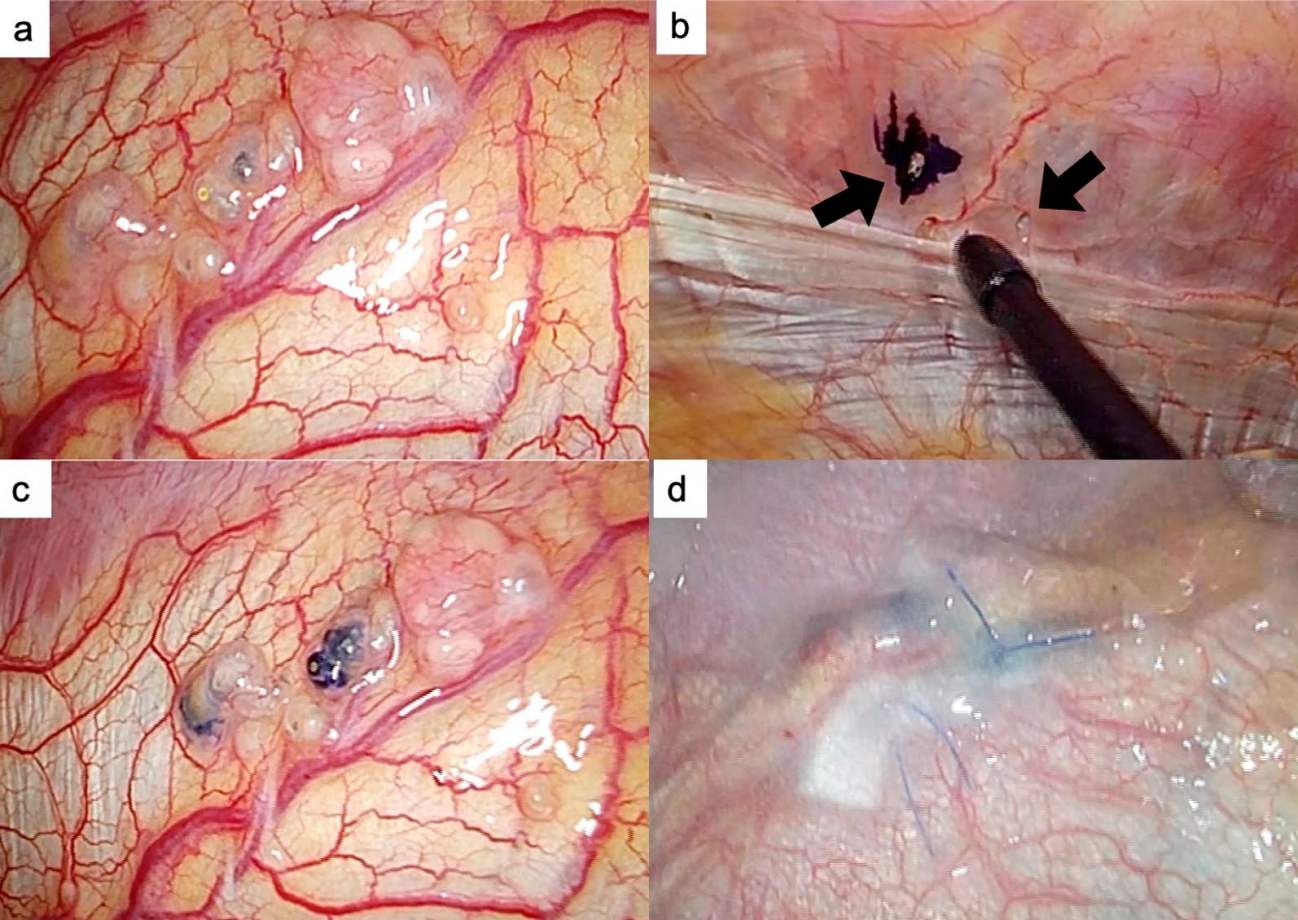
Surgical findings of case 4. **a** Thinned diaphragm and cystic lesions were detected at the central tendon of the diaphragm. No pores or bubbles due to laparoscopic pneumoperitoneum were observed on the diaphragm on the thoracic side. **b** Laparoscopic view of the diaphragmatic fistulas. Two small fissures with a diameter of 1 mm were detected on the abdominal side; we soaked the dye containing indigo carmine into the pores in the peritoneal cavity. **c** Two lesions with blue staining were noted on the diaphragm from the thoracic side. **d** Each lesion was closed with a 4–0 non-absorbable monofilament and Teflon pledget, with single U suture. Furthermore, the lesions were covered with a sheet of absorbable polyglycolic acid felt and sprayed with fibrin glue on the thoracic side

Cases 3 and 4 had no fistula and no sign of bubbles on thoracoscopy; therefore, we inserted a laparoscope into the abdominal cavity. In case 3, there was no fistula responsible for the PPC on the diaphragm, and we only reinforced the diaphragm with PGA sheets and fibrin glue. In case 4, the thinned diaphragm and some cystic lesions were observed at the central tendon of the diaphragm, but we failed to detect any pores or bubbles caused by pneumoperitoneum. On laparoscopy, however, two pores were detected on the diaphragm; therefore, we soaked indigo carmine in the pores of the peritoneal cavity. Thoracoscopy revealed cystic lesions with blue staining of the diaphragm. The lesions were closed by suturing with a Teflon pledget and reinforced using the same procedure (Fig. 4). In this case, the diaphragm was so thin that we used Teflon pledget to avoid tearing the diaphragm.

In all cases, a 20-Fr silicone drain (silicone thoracic catheter, NIPRO, Osaka, Japan) was inserted into the right pleural cavity. The chest tube was removed after confirming that pleural effusion did not increase after resuming CAPD. In case 1, CAPD catheter infection was noticed after surgery; this improved with catheter replacement and antibiotic administration. Infection from the abdominal port could not be ruled out; however, the distance between the abdominal port and the CAPD catheter was approximately 7 cm. Thus, the possibility of infection might be low. No recurrence of PPC was noted in any of the cases, and CAPD was continued. The duration of restarting CAPD for each case was 19, 7, 8, and 8 days, and the postoperative stay was 36, 11, 15, and 16 days, retrospectively. The postoperative observation period for each case was 665, 115, 334, and 198 days, respectively (Table 1).

## Discussion

PPC is defined as various porous syndromes caused by the passage of pleural effusion or blood through the pleural and abdominal cavity [1]; it is rarely observed in patients with CAPD, liver cirrhosis, or ascites due to malignant tumors. It appears on the right side of the thorax in approximately 90% of cases [2, 3]. The exact mechanisms of PPC development are not well understood, but it is believed that abnormalities of the diaphragm and/or breakdown of the lymphatic network lead to pleural effusion [4–6]. Anatomical defects are more common in the right diaphragm; the left side of the diaphragmatic defects is covered with the heart and pericardium, and there is more lymphatic network on the right side than on the left [2]. Additionally, the lateral edge of the central tendon has abundant lymphatic stomata [7]. These factors may explain the preponderance of right-sided diaphragmatic fistulas.

Edward and Ungar first reported PPC under CAPD in 1967 [8] and in 1996, Di Bisceglie et al. [9] reported the first case of VATS performed for PPC. Since then, there have been several reports on the effectiveness of VATS. VATS (1) leads to early resumption of CAPD after surgery; (2) has a high intraoperative diagnosis rate and treatment results; (3) has a high safety threshold and can be performed even in patients with end-stage renal disease or hepatic failure; and (4) needs a pathological examination to confirm other causes (e.g., congenital defects, tumor, infection, or amyloidosis) for the pathogenesis of PPC [10–12]. However, no standard method is available for the intraoperative diagnosis or surgical procedures for PPC.

Saito et al. reported a success rate of 89% in fistula-confirmed cases but only 38% in fistula-unconfirmed cases [11]. Thus, it is important to detect fistulas responsible for PPC during surgery for the successful treatment of PPC. To our knowledge, four intraoperative diagnostic methods have been reported: careful observation in the pleural cavity; dye injection (indigo carmine or indocyanine green [13]); pneumoperitoneum[14]; and laparoscopic observation [15]. Certain other methods can identify the location of fistulas without a laparoscopic port during surgery [16, 17].

Compared to these methods, the laparoscopic approach has a risk of peritonitis, forcing patients to suspend CAPD, or to switch to hemodialysis. However, our method is technically simple and enables pneumoperitoneum, laparoscopic observation, and repair of the diaphragm from the abdominal side. It can also be particularly effective in cases of severe pulmonary diaphragmatic adhesion, such as PPC recurrence. Yorinaga et al. [15] reported that the shape of the fistula, a fissure, observed with a laparoscope, may be related to the detection rate of the lesions with a thoracoscope, and the same findings were observed in our case 4. Additionally, the half-lateral decubitus position facilitated the operation from the abdominal side, and even when the operation was performed from the thoracic side, the bed could be rotated appropriately to allow facilitation.

As a surgical procedure to treat PPC, the following methods have been reported for repairing fistulas, either singly or in combination: direct suture or ligation; excision with a stapler [14]; covering with PGA sheets with or without fibrin glue [15]; pericardial fat pad tissues [18]; a pedicled latissimus dorsi muscle flap, or a falciform ligament [19]. However, the superiority of each treatment is unclear. A previous study reported that the stapling line was torn after suturing the diaphragm with a stapler [20]. This could probably be due to the pressure effect on the top and edge of the stapling line, particularly after resumption of CAPD. Thus, we prefer direct sutures. There might not be a problem with any particular suture method (Z sutures, horizontal mattress sutures, etc.); however, direct suture could tear fragile tissues and may be better used together with other reinforcements, such as a Teflon pledget, depending on the diaphragm thickness, as in case 4.

Furthermore, in PPC, fistulas are frequently found in the center of the diaphragm tendon [12], which should be focused on when observing the diaphragm. Therefore, for cases such as case 3 in which we failed to detect a fistula, it is important to reinforce the center of the diaphragm tendon to prevent recurrence.

Based on the strategy shown in Fig. 1, we combined VATS with a laparoscopic approach and treated the fistulas. Our methods need a laparoscopic port, but enabled observation and repair from the abdominal cavity side. In three of the four cases, diaphragmatic fistulas could be identified and closed. We would like to increase the number of PPC cases and further examine the usefulness of this approach.

## Conclusion

We herein report four cases in which thoracoscopic and laparoscopic approaches were effective for inspecting and treating fistulas responsible for PPC. For accurate diagnosis and treatment, we recommend VATS combined with a laparoscopic approach as a surgical procedure for PPC.

## Data Availability

Not applicable.

## Abbreviations

PPC: Pleuroperitoneal communication
CAPD: Continuous ambulatory peritoneal dialysis
VATS: Video-assisted thoracic surgery
PGA: Absorbable polyglycolic acid
